# Improved Locomotor Recovery in a Rat Model of Spinal Cord Injury by BioLuminescent-OptoGenetic (BL-OG) Stimulation with an Enhanced Luminopsin

**DOI:** 10.3390/ijms232112994

**Published:** 2022-10-27

**Authors:** Ebenezer C. Ikefuama, Griffin E. Kendziorski, Kevin Anderson, Lateef Shafau, Mansi Prakash, Ute Hochgeschwender, Eric D. Petersen

**Affiliations:** 1 Program in Neuroscience, Central Michigan University, Mount Pleasant, MI 48859, USA; 2 College of Medicine, Central Michigan University, Mount Pleasant, MI 48859, USA

**Keywords:** optogenetic, bioluminescence, spinal cord injured (SCI), non-invasive, stimulation, chemogenetic, neuromodulation

## Abstract

Irrespective of the many strategies focused on dealing with spinal cord injury (SCI), there is still no way to restore motor function efficiently or an adequate regenerative therapy. One promising method that could potentially prove highly beneficial for rehabilitation in patients is to re-engage specific neuronal populations of the spinal cord following SCI. Targeted activation may maintain and strengthen existing neuronal connections and/or facilitate the reorganization and development of new connections. BioLuminescent-OptoGenetics (BL-OG) presents an avenue to non-invasively and specifically stimulate neurons; genetically targeted neurons express luminopsins (LMOs), light-emitting luciferases tethered to light-sensitive channelrhodopsins that are activated by adding the luciferase substrate coelenterazine (CTZ). This approach employs ion channels for current conduction while activating the channels through treatment with the small molecule CTZ, thus allowing non-invasive stimulation of all targeted neurons. We previously showed the efficacy of this approach for improving locomotor recovery following severe spinal cord contusion injury in rats expressing the excitatory luminopsin 3 (LMO3) under control of a pan-neuronal and motor-neuron-specific promoter with CTZ applied through a lateral ventricle cannula. The goal of the present study was to test a new generation of LMOs based on opsins with higher light sensitivity which will allow for peripheral delivery of the CTZ. In this construct, the slow-burn *Gaussia* luciferase variant (sbGLuc) is fused to the opsin CheRiff, creating LMO3.2. Taking advantage of the high light sensitivity of this opsin, we stimulated transduced lumbar neurons after thoracic SCI by intraperitoneal application of CTZ, allowing for a less invasive treatment. The efficacy of this non-invasive BioLuminescent-OptoGenetic approach was confirmed by improved locomotor function. This study demonstrates that peripheral delivery of the luciferin CTZ can be used to activate LMOs expressed in spinal cord neurons that employ an opsin with increased light sensitivity.

## 1. Introduction

Spinal cord injury (SCI) is considered one of the most devastating events in someone’s life, which frequently leads to perpetual paralysis below the site of injury with limited treatment options [1]. SCI arises from primary and secondary injury mechanisms. Primary injury indicates immediate physical damage to the spinal cord emanating from the contusion, concussion, compression, contraction, shear, and laceration of the neural tissue [2]. After some minutes following a primary injury, secondary injury is triggered, and it involves changes in the local ionic concentrations, loss of regulation of local and systemic blood pressure, reduced spinal cord blood flow, breakdown of the blood–brain barrier, penetration of serum proteins into the spinal cord, free radicals/lipid peroxidation production, inflammatory responses (alterations in cytokines and chemokines), apoptosis, excitotoxicity, calpain proteases, neurotransmitter aggregation, and imbalance of activated metalloproteinases [3,4,5]. These changes invariably lead to ischemia, edema, hypoxia, loss of myelin, necrosis, apoptosis of spinal cord tissue, glial cell proliferation, and the disconnection of living neurons, culminating in the formation of a microenvironment that is unfavorable for nerve regeneration [6,7,8,9].

To date, no effective therapy exists for complete axon regeneration after SCI. Over the past decade, significant progress has been made not only in traditional research fields, such as inflammation, scar formation, cell transplantation, axon regeneration, and biomaterial repair, but also in determining the mechanisms of spinal cord automation, spontaneous circuit reorganization, and functional recovery after SCI [10,11].

**Figure 1:**
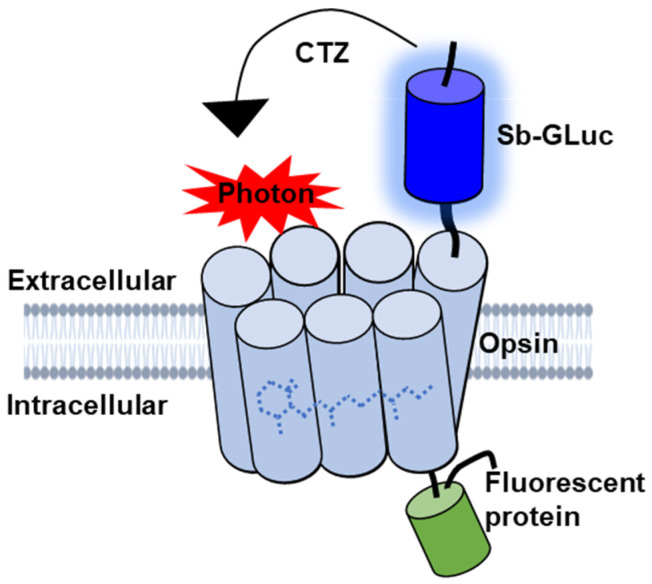
Schematic diagram of the genetically engineered LMO. The luciferase slow-burn Gaussia (sbGLuc) is tethered to the opsin CheRiff and the green fluorescent protein (EGFP).

BioLuminescent-OptoGenetics (BL-OG) uses optogenetic elements that are activated by light generated by a luciferase tethered to a light-sensitive channelrhodopsin (luminopsin, LMO) [12]. The bioluminescent light is produced by the oxidation of a specific enzymatic substrate, the luciferin coelenterazine (CTZ). Stimulation only occurs when the CTZ is present, producing bioluminescent light through catalysis by the luciferase, resulting in the activation of the opsin (Figure 1). This approach takes advantage of both opto- and chemogenetic concepts by utilizing ion channels for current conduction while activating the channels through the application of a chemical compound, thus allowing non-invasive stimulation and recruitment of all targeted actuators as opposed to only those that can be reached by light from a physical source [12,13,14,15,16,17,18]. Moreover, bioluminescence is light emitted without heat (“cold light”), and hence does not produce the damage encountered for physical light [19,20,21,22,23]. Utilizing LMOs for neural stimulation in the spinal cord presents an innovative approach for activating neurons that can be therapeutically beneficial to recovery following SCI that was not previously possible with other techniques.

In this study, we sought to stimulate neurons that lie below the injury epicenter without any implanted hardware to strengthen spared neuronal connections through the activation of light-sensitive ion channels. Upon the opening of the ion channel, there is the influx of sodium ions (Na^+^) and efflux of potassium ions (K^+^) leading to excitatory postsynaptic potential and neuronal depolarization. 

Prior studies have shown that neuronal activation using BL-OG improves hindlimb motor function following a severe thoracic contusion SCI [24]. Our initial study used an intraventricular cannula to deliver CTZ directly into the cerebrospinal fluid through a chronic injection port. By using an LMO based on CheRiff (LMO3.2), a fast and sensitive blue-shifted opsin from *Scherffelia* [25], to replace the *Volvox* channel used in LMO3, this study tested if a peripheral, intraperitoneal injection of CTZ combined with our new LMO would produce beneficial outcomes following SCI.

## 2. Results

### 2.1. LMO3.2 Induces Higher Photocurrents Compared to LMO3

**Figure 2:**
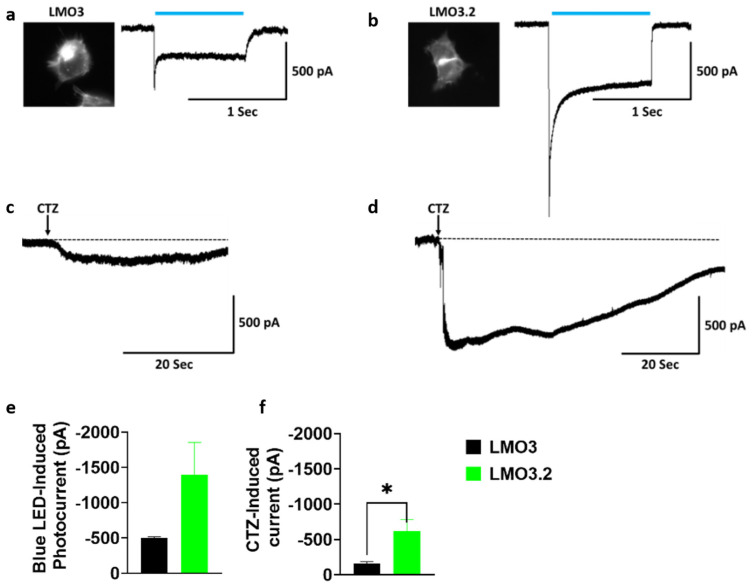
Comparison of LMO3 and LMO3.2 activation using blue light and CTZ. (**a**) Left, EYFP fluorescence in HEK cells expressing LMO3. Right, representative photocurrent after blue light stimulation. (**b**) Left, EGFP fluorescence in transiently lipofected HEK cells expressing LMO3.2. Right, representative photocurrent after blue light stimulation. (**c**) Representative photocurrent after activation of VChR1 based LMO3 via CTZ-induced bioluminescence emission from sbGLuc. (**d**) Representative photocurrent after activation of CheRiff based LMO3.2 via CTZ-induced bioluminescence emission from sbGLuc. (**e**) Blue light induces a greater photocurrent in LMO3.2-expressing cells than LMO3. (**f**) CTZ induced bioluminescence results in a significantly greater photocurrent in LMO3.2-expressing cells than LMO3; * *p* < 0.05 versus LMO3 (Student’s *t* test). Error bars indicate SEM; *n* = 3 cells each.

To compare the response to blue light or CTZ-induced photocurrent activation to our previous generation of LMOs, we transfected HEK293 cells with LMO3 and LMO3.2. Whole-cell patch clamp recordings revealed a large inward photocurrent in LMO3.2-expressing cells (−1397.9 pA) than in LMO3-expressing cells (−502.9 pA) after stimulation with blue light (Figure 2a,b,e). Similarly, CTZ induced a much larger response that was 4-fold higher in LMO3.2 (−621.9 pA) compared to LMO3 (−159.9 pA) (Figure 2c,d,f).

### 2.2. Intraperitoneal Injection of CTZ Leads to Measurable Bioluminescence Emission

**Figure 3:**
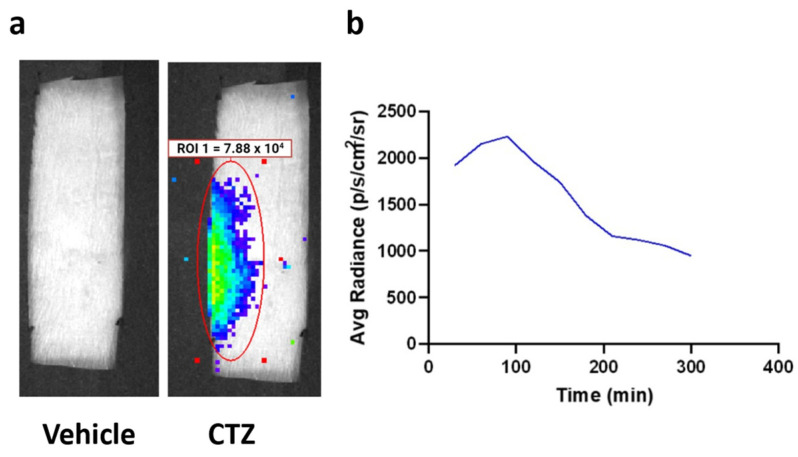
Bioluminescence emission from LMO3.2 expressing rats treated with either vehicle or CTZ. (**a**) In vivo bioluminescent imaging reveals luminescence concentrated over the lumbar spinal cord. Peak luminescence can be seen in the pseudo-colored yellow/green region directly over the spinal cord. (**b**) Average luminescence in the region of interest over the spinal cord. Radiance peaked between 60 and 120 min before slowly dissipating.

IP injection of CTZ generated bioluminescence that was observed over the lumbar region of the spinal cord (Figure 3a). Peak bioluminescence was detected between 60 and 120 min after CTZ treatment, demonstrating the time of highest neuronal stimulation (Figure 3b). Furthermore, in vivo bioluminescent imaging verified viral expression, underscoring BL-OG’s dual function as a cellular activity modifier and self-reporter. 

### 2.3. Rats That Received Stimulation via LMO3.2 Showed Improved Locomotor Function after SCI

**Figure 4:**
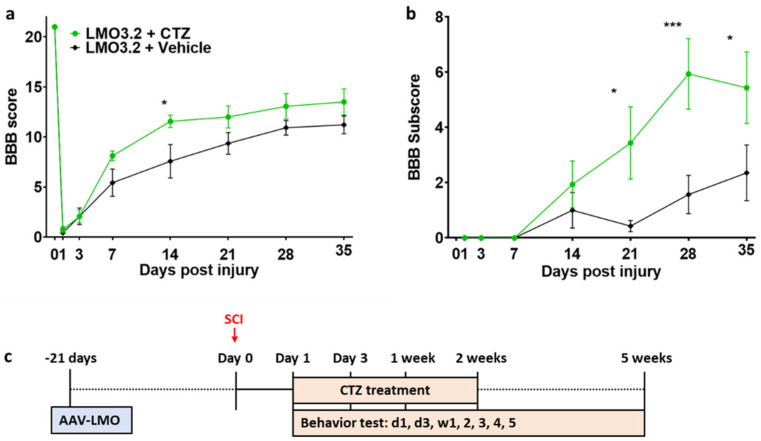
Rats expressing the CheRiff-containing LMO3.2 and treated with CTZ showed improved locomotor function after SCI. BBB locomotor rating score (**a**) and subscore (**b**) were performed to assess functional recovery of rats’ hind limbs at Day 1, Day 3, Week 1, Week 2, Week 3, Week 4, and Week 5 following spinal cord injury. LMO3.2 + vehicle (*n* = 7) and LMO3.2 + CTZ (*n* = 8). LMO3.2 + CTZ vs. LMO3.2 + vehicle: * *p* < 0.05, *** *p* < 0.001. Error bars ± standard error of mean (SEM). (**c**) Timeline for experimental procedures. The virus was injected three weeks before spinal cord injury with CTZ injections every other day for two weeks following the injury. Behavior testing was conducted starting the day after the injury and for five weeks post-injury.

CTZ administration led to improved locomotor function in the LMO3.2-expressing rats compared to the vehicle-treated group (Figure 4). The high BBB scores on Week 1 and Week 2 coincide with the CTZ treatment window. At Week 5, the LMO3.2 + CTZ group had a mean BBB score of 13.5 (Figure 4a), which represents rats that can make consistent weight-supported plantar steps; consistent front–hindlimb coordination and predominant paw position during locomotion are rotated (internally or externally) when making initial contact with the surface as well as just before lifting off at the end of stance. The vehicle-treated animals had a mean BBB score of 11.2, representing frequent to consistent weight-supported plantar steps with no front–hindlimb coordination. The ANOVA analysis of BBB scores showed a significant effect for time (F (7, 91) = 202.69, *p* < 1.0 × 10^−15^) and an interaction effect for treatment by time point (F (7, 91) = 2.681721, *p* = 0.014) and (F (1, 13) = 3.28, *p* = 0.093) for main effect for treatment. Similarly, LMO3.2 + CTZ-treated rats performed better on the BBB subscores with a mean of 5.44 at the end of the experiment. The control rats had a mean subscore of 2.36 (Figure 4b). The ANOVA analysis of BBB subscores showed a significant effect for time (F (6, 78) = 15.13660, *p* < 2.07 × 10^−11^) and a significant main effect for treatment (F (1, 13) = 5.117970, *p* = 0.041). There was also a significant interaction effect for treatment by time point (F (6, 78) = 4.257434, *p* = 0.00092). These results indicate that stimulation with IP-administered CTZ in rats expressing LMO3.2 recover faster and to a greater extent than vehicle-treated animals. 

### 2.4. CatWalk Automated Gait Analysis Detected Difference in Stand between Vehicle- and CTZ-Treated Rats

**Figure 5:**
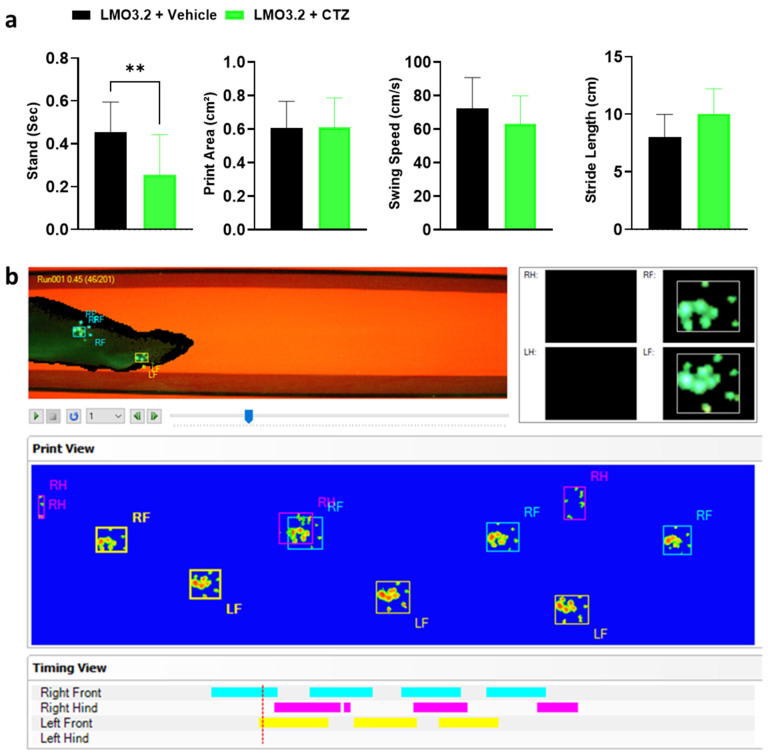
Gait parameters assessed and graphical print view of an injured rat on the CatWalk. (**a**) Stand, print area, swing speed, and stride length of vehicle- or CTZ-treated spinal cord injured rats. Stimulated rats showed a significant decrease in stand but no improvement in all other parameters analyzed. LMO3.2 + vehicle (*n* = 7) and LMO3.2 + CTZ (*n* = 8). LMO3.2 + CTZ vs. LMO3.2 + vehicle: ** *p* < 0.001. Error bars ± standard error of mean (SEM). (**b**) Pawprints were acquired using the CatWalk XT automated gait analysis system. Inappropriately labeled prints were manually corrected frame by frame for each run following auto-classification on the CatWalk XT^®^ software, version 10.6, Noldus Information Technology, Wageningen, The Netherlands.

CatWalk (Noldus Information Technology) generates multiple gait parameters, and based on prior studies with SCI, we focused on the following four key gait parameters: stand, print area, swing speed, and stride length. The stand is defined as the duration in seconds of contact of a paw with the glass plate. A higher stand duration indicates impairment in gait function [26]. We observed a significant difference in stand between the CTZ- and vehicle-treated animals (t = 3.272, df = 28, *p* = 0.0028). The vehicle-treated rats had significantly greater stand times compared to CTZ-stimulated rats (Figure 5a). An increased print area indicates improved fine digit motor function and trunk stability, and in this study, treatment and vehicle groups placed almost equal pressure on their hindlimbs (t = 0.02212, df = 28, *p* = 0.9825). For swing speed, which provides information on the locomotion and muscle tone of the limbs, we observed no difference between groups (t = 0.3711, df = 28, *p* = 0.7134). Finally, the two groups had a comparable distance between successive placements of the same paw (stride length) (t = 0.6825, df = 28, *p* = 0.5005). An increase in the stride length following an injury indicates greater trunk stability and suggests better improvement of coarse muscle strength [27].

### 2.5. Experimental and Control Groups Were Comparable Regarding Viral Transduction and Severity of Contusion Injury

**Figure 6:**
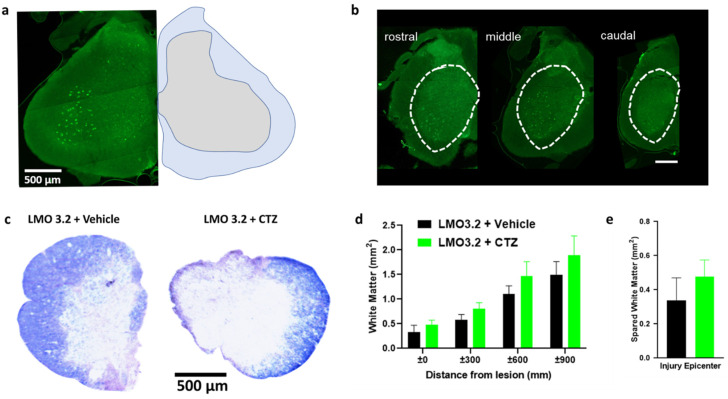
AAV-hSyn-LMO3.2-EGFP expression, eriochrome cyanine staining and quantification of spared white matter around and within the injury epicenter. (**a**) AAV-hSyn-LMO3.2-EGFP expression (**left**) and anatomical annotations (**right**). (**b**) There is robust viral transduction of the lumbar region of the spinal cord. (**c**) Eriochrome cyanine staining shows similar loss of white matter within the injury epicenter of vehicle- and CTZ-treated groups. (**d**,**e**) All animals were uniformly injured and there was no statistically significant difference between the two groups. Data are presented as mean ± SEM.

We found robust GFP expression in the ventral horn of the spinal cord when using the pan-neuronal hSyn promoter (Figure 6a). The spread of viral transduction at the injection site was around 2 mm rostral to caudal (Figure 6b) and was comparable between animals. The extent of white matter loss can be assessed at the site of injury by using eriochrome cyanine, a stain for myelin. We confirmed extensive damage to the white matter at the epicenter of the injury (Figure 6c) consistent with this SCI model. There was no significant difference between the treatment and vehicle groups (Figure 6d,e). This confirms that lumbar stimulation following injury does not appear to affect degeneration and is consistent with our prior study [24].

## 3. Discussion

Our results confirm that non-invasive modulation of the activities of neurons in the spinal cord led to significantly improved locomotor function after SCI, comparable to our previous study. Here, we used LMOs with optogenetic elements of increased light sensitivity and observed that systemic, peripheral delivery of CTZ led to corresponding outcomes compared to delivery via a lateral ventricular cannula following a severe SCI. Neurons expressing LMO 3.2 below the site of the injury were activated by the bioluminescent light generated from the enzymatic breakdown of CTZ. In this study we were able to take advantage of the ultra-low light sensitivity of the excitatory opsin CheRiff to enable peripheral delivery of the CTZ, which resulted in improved locomotor recovery compared to vehicle-treated animals. 

Through this study, we were able to surmount most critical limitations associated with traditional optogenetics and electrical stimulation, which include invasiveness of a chronic implant, increased risk of infection to the patient, potential formation of a glial scar around implanted electrodes, degradation of the electrodes after placement, and off-target activation of cells that lie around the electrode [28,29]. In a previous study, we demonstrated that neural activation of lumbar spinal neurons using BioLuminescent-OptoGenetic stimulation can significantly improve hind limb motor function [24]. Nonetheless, this prior work still required the use of cannulas implanted into the lateral ventricle cannula for application of the luciferase substrate. We overcame this limitation by utilizing a new LMO based on a more sensitive optogenetic channel which requires lower dosing of the substrate for activation, making peripheral delivery via IP feasible.

The findings and importance of our study are further strengthened by other previously published reports. For instance, optical stimulation was used to selectively activate hind limb muscles in a rodent model of SCI by intra-muscular injection of the adeno-associated viral vector carrying ChR-2 and a motor-neuron-specific promoter [30]. Furthermore, Hägglund and colleagues optically stimulated spinal interneurons in a transgenic mouse line expressing ChR-2 channels, and results showed rhythmic activation of selective muscles responsible for locomotion [31]. Deng and colleagues reported a similar improvement in locomotor function in mildly injured rats following optogenetic stimulation of pyramidal neurons in the M1 region of the cortex, where stimulation of M1 cortical neurons will invariably target spinal neurons that lie above the injury epicenter [32]. Although very promising, transduction of this group of neurons will present a challenge, since this method requires a craniotomy for the delivery of an AAV vector to the brain. To bring this approach closer to the clinic, a better option would be to inject the AAVs into the spinal cord during corrective surgery to alleviate spinal cord stenosis, remove damaged tissues such as broken bones, and restore posture. It is pertinent to point out that our study differs from the above studies in the mode of activation of the optogenetic actuators, duration of stimulation, targeted neuronal population, and severity of injury. We employed a severe form of SCI and neurons below the level of the injury were exclusively activated for two weeks by using highly light-sensitive opsins and biological light instead of a physical light source. Recently, BL-OG stimulation was demonstrated to promote regeneration of axons following peripheral nerve injury [33]. This study highlights the potential for BL-OG to promote recovery via inducing neuronal plasticity, which is also consistent with the findings of our previous SCI study. 

To further explore if there are significant improvements in locomotor behavior, we used in addition to BBB scoring the CatWalk XT gait analysis system as an objective technique. Different from the BBB scoring system, this is a very sensitive assessment method, and the results revealed a significant decrease in the stand time for the treatment group but did not reveal differences in other parameters in the LMO3.2 + CTZ-treated animals. Here, the rats that received stimulation applied little pressure on the hindlimbs, while undertaking weight-supported plantar steps. Due to the severity of injury in our SCI model, we were unable to fully take advantage of the complexity of the CatWalk analysis. Many animals in the vehicle group were unable to walk using their hindlimbs, leading to missing data points. This could be the reason why no differences were detected in the other parameters assessed and why it was not possible to combine different CatWalk gait parameters to use all available analysis [26].

In summary, our study demonstrates that peripheral injection of the luciferin CTZ can be used with LMOs expressed in spinal cord neurons that employ an opsin with increased light sensitivity. Activation of LMO3.2 following a severe thoracic-level contusion injury resulted in ameliorated motor deficits. This is evidenced by the increase in BBB scores and BBB subscores and the ability of the LMO3.2 + CTZ rats to take weight-supported plantar steps.

### 3.1. Relationship to Previous Studies

We previously showed the efficacy of BL-OG in improving locomotor recovery following severe spinal cord contusion injury in rats by expressing the excitatory LMO3 under the control of pan-neuronal (hSyn) and motor-neuron-specific (Hb9) promoters below the injury site; CTZ was delivered through a lateral ventricle cannula [24]. To avoid the invasiveness of the intraventricular cannula and bring this approach closer to clinical application, we developed LMOs which are highly light sensitive and that will require lower levels of luciferin/substrate for optimal activation. Through intraperitoneal delivery of CTZ, we achieved results in locomotor recovery comparable to the use of a lateral ventricular cannula.

### 3.2. Limitations of the Study

Despite the positive outcome of this study, there are several limitations. First, we targeted all neurons below the injury site by using the synapsin promoter instead of a specific promoter to target a subset of neurons. Future experiments will use neuron-subtype-specific promoters to more selectively stimulate specific subsets of neurons after SCI. Furthermore, as new and better light-emitting luciferases and also luciferins that are highly efficient at crossing the blood–brain barrier are being developed, it will be promising to test these new bioluminescent components and subsequently apply them in preclinical SCI studies. In follow-up studies we also plan to test stimulation in the chronic phase of SCI, as this treatment timeline will be more translatable to SCI patients.

Together, our study provides further evidence and emphasizes that stimulating LMO-expressing neurons through intraperitoneal delivery of CTZ enhances motor function recovery and has potential as a therapeutic option for SCI patients. 

## 4. Materials and Methods

### 4.1. Animals

Sixteen (16) adult female Sprague Dawley rats (*n* = 8 per group), weighing 250–370 g (g) and 4–6 months of age, bred on-site, were used. All experimental procedures were performed following the National Institutes of Health (NIH) guidelines and were approved by the Institutional Animal Care and Use Committee (IACUC) of Central Michigan University. Animals were kept in 12 h light/dark cycle rooms and fed ad libitum. 

### 4.2. Plasmids and Viruses

The optogenetic actuator, *Scherffelia dubia* channelrhodopsin (sdChR), was identified while screening for plant genomes [34,35]. sdChR showed promising sensitivity and a blue activation spectrum (peak excitation wavelength λmax = 474 nm). Cohen and colleagues improved membrane targeting and also shifted the activation spectrum to λmax = 460 nm by introducing the trafficking sequence from Kir2.1 and mutation E154A, respectively; this final construct was named CheRiff [25]. The transgenes of CheRiff, the light-emitting moiety, slow-burn Gaussia luciferase (sbGluc), and the fluorescent reporter protein enhanced green fluorescent protein were subcloned into an adeno-associated virus (AAV) plasmid under the human synapsin I promoter’s control, pAAV/hSyn, to obtain pAAV-hSyn-sbGLuc-CheRiff-EGFP (Addgene plasmid # 114108). For a detailed description of the generation of LMO viral vectors, see Berglund et al., 2016 [12,36]. 

### 4.3. Electrophysiological Recordings from HEK293 Cells

LMO3.2 was characterized similarly to our previous LMO variants as described in [12,15,36]. Briefly, transiently lipofected HEK293 cells for LMO3 and LMO3.2 were seeded at low density on 15 mm poly-D-lysine coated glass coverslips in 12-well plates and incubated for 24–48 h prior to electrophysiological recording. A coverslip was transferred to a recording chamber (RC26-GLP, Warner Instruments, Holliston, MA, USA) mounted on an upright microscope (BX51WI, Olympus, Tokyo, Japan) and perfused with ACSF (1–1.5 mL/min) containing (in mM): 150 NaCl, 3 KCl, 10 HEPES, 2 CaCl_2_, 2 MgCl_2_, and 20 D-glucose (pH 7.4, ~300 mOsm/kg) at 35 ± 1 °C recording temperature. The intracellular solution contained (in mM): 130 K+-gluconate, 8 KCl, 15 HEPES, 5 Na2-phosphocreatine, 4 Mg-ATP, 0.3 Na-GTP (pH 7.25, Osm 300 mOsm/kg). Borosilicate glass micropipettes were manufactured using a vertical puller (PC-100, Narishige, Tokyo, Japan) and had resistances from 4–6 MΩ. Cells expressing EYFP or EGFP were visually targeted using epifluorescence. VChR1 was excited through the objective (LUMPLFLN40XW, NA 0.8, Olympus) using a metal halide light source (130 W, U-HGLGPS, Olympus) together with filter cube for blue (Ex/Em: 480/530 nm, U-MNIBA3, Olympus) excitation. An electronic shutter (Lambda SC, Sutter Instruments, Novato, CA, USA) was used to program the time window of excitation. Photocurrents were evoked by 1 s exposure to blue light at irradiances of 30 mW/cm^2^. Whole-cell voltage clamp recordings were performed at −60 mV using a Multiclamp 700b amplifier and Digidata 1440 digitizer together with the pCLAMP 10 recording software (Molecular Devices, San Jose, CA, USA). After break-in, the photocurrent response of the cell was determined using a gap-free protocol followed by application of CTZ (250 μM) through the perfusion (final concentration in bath ~100 μM). The total number of LMO3- and LMO3.2-expressing HEK cells used in recordings were 3 for each construct. Data were sampled at 10 kHz, filtered at 3 kHz, and analyzed in Clampfit 10.7 (Molecular Devices).

### 4.4. Surgery

All surgeries were performed with isoflurane anesthesia under aseptic conditions. Thermal support was provided via a heat plate, and a toe pinch was applied to monitor the depth of anesthesia. 

### 4.5. Viral Injections

The animals were mounted on a stereotaxic frame, the dorsal surface was shaved, disinfected with a chlorohexidine solution, and a cutaneous incision (3 cm) was performed to expose the backbone. T13 and L1 vertebrae were identified, and dorsal and intervertebral muscles were cleared away to expose the vertebral column. The column was stabilized using vertebral clamps. Bone was removed to expose the spinal cord. The virus was injected using a 10 µL World Precision Instruments syringe with a 35 G beveled needle. The virus was bilaterally injected 0.5 mm lateral to the midline and 1.5 mm ventral to the surface. Both injection points received 2.5 µL at 5 × 10^12^ copies/mL of AAV-hSyn-sbGLuc-CheRiff-EGFP. Injections were performed at 0.16 µL/min and left in place for 5 min following the injection to prevent backflow (Figure 4). 

### 4.6. Spinal Cord Injury

Three weeks following viral injections, a laminectomy was performed at the level of T-9. The spinal cord was stabilized using vertebral clips. A severe contusion was induced with a New York University Impactor device with a weight of 10 g dropped from a height of 25 mm [37]. Following the contusion injury, the Crede maneuver [38] was performed to empty the bladder: rats were held upright, and gentle pressure was applied on the protruding bladder from top to bottom. This was performed four times per day for two days and then twice a day until self-voiding returned. 

Following injections or injury, muscle tissue was sutured with 4–0 Silk sutures (Oasis, Mettawa, IL, USA). The skin was closed with staples, and the wound site was covered with antibiotic ointment. Subcutaneous buprenorphine (0.02 mg/kg) and meloxicam (1.0 mg/kg) were delivered to animals twice daily for two days after all procedures.

### 4.7. IVIS Imaging

Animals were anesthetized with isoflurane in an induction chamber, CTZ was administered IP, and rats were immediately placed inside the IVIS Lumina LT (Perkin Elmer, Waltham, MA, USA). Anesthesia in the IVIS chamber was maintained at a flow rate of 1.5–2%. Images were taken with an exposure of 30 min, f-stop at 1, with large binning, over two hours. Only animals with confirmed expression of LMOs were used in the study.

### 4.8. Stimulating Treatment

Animals received IP injections of water-soluble CTZ (Nanolight #3031) or vehicle solution (vehicle; Nanolight #3031C). Treatment was administered every other day, beginning the day after the SCI and lasting for two weeks (Figure 4c), following the same treatment timeline as our previous study. Treatment with CTZ was given IP at 2 mg/kg. On days on which animals received treatment as well as behavioral testing, the treatment was always given following the behavioral tests.

### 4.9. Assessment

#### 4.9.1. Basso, Beattie, and Bresnahan (BBB) Open-Field Locomotion Score

The BBB scale was used to score hind limb motor function. Rats were scored from 0–21, with 0 being complete paralysis and 21 being perfect gait (Figure 4). At a score of 10, the animals begin to take their first weight-bearing steps [39]. All tests were conducted by two observers. One rat was excluded from the vehicle-treated group and from subsequent analysis due to insufficient injury (BBB score > 1 on day 1). 

#### 4.9.2. BBB Subscore

Animals were also scored on the BBB subscale, which looks at variation and functional deficits between limbs as well as toe clearance, trunk stability, and tail position. This measure is specifically helpful in moderate to severe SCI [40,41]. 

#### 4.9.3. CatWalk Assessment

At the end of the testing period, gait analysis was performed using the CatWalk XT gait analysis system (version 10.6; Noldus Information Technology, Wageningen, The Netherlands). Briefly, light from a fluorescent tube is sent through a glass plate. Light rays are completely reflected internally. Once the rat’s paw is in contact with the glass surface, light is reflected downwards, forming a sharp image of a bright paw print. This image is captured by a camera placed under the glass floor (Figure 5b). We focused this assessment on the hindlimb performance under the following dependent variables: stand, print area, stride length, and swing speed. Rats that could not run or those with missing data were assigned zero (0) in print area, stride length, and swing speed. The maximum value of the right or left hindlimbs was substituted for the missing right/left stand. We postulate that SCI would diminish all measures towards 0 except for stand, which would approach infinity due to a complete lack of movement. Finally, the average of all trials per animal was used for further analysis.

#### 4.9.4. Viral Expression and Eriochrome Cyanine Staining

At the end of the 5-week testing period, spinal cord injured rats were deeply anesthetized with carbon dioxide and transcardially perfused with cold phosphate-buffered saline (PBS) followed by 4% *w*/*v* paraformaldehyde solution. Spinal cords were removed and post-fixed for two days then placed in 30% sucrose for cryoprotection, and then flash-frozen in isopentane (2-methyl butane) and stored at −80 °C [42]. Lumbar regions were embedded in M1 embedding matrix (Richard-Allan Scientific LLC, Kalamazoo, MI, USA), cryosectioned at 30 μm using CryoStar NX50 (Thermo Fisher, Waltham, MA, USA), and serially mounted on pig-gel-coated slides to check EGFP expression and to perform eriochrome cyanine staining. To quantify the level of spared white matter across the experimental animals, we performed eriochrome cyanine (EC, Sigma, Darmstadt, Germany) staining [24]. Briefly, thoracic sections mounted on slides were air-dried, dehydrated, and defatted in graded ethanol solutions (50, 70, 90, 95, 100%, 3 min each), followed by xylene (10 min), rehydrated in graded ethanol solutions, then incubated in EC solution for 10 min. Slides were rinsed twice with water, differentiated in 0.5% ammonium hydroxide, and then rinsed twice with water [43]. Slides were dehydrated in graded ethanol solutions to xylene and coverslipped with Eukitt mounting media (Sigma). Slides were scanned with a Nikon Coolscan IV slide scanner (Nikon, Tokyo, Japan). We quantified the spared white matter by tracing the blue-stained area using ImageJ software (version 1.53q, U.S. National Institutes of Health, Bethesda, MD, USA).

### 4.10. Statistical Analysis

For all behavioral and histological scoring analysis, researchers were blind to condition. All of the data were expressed as the mean ± standard error of the mean (SEM). The statistical significance for the patch data, BBB scores and subscores, CatWalk, and eriochrome cyanine data were determined by *t*-test, and one-way or two-way analysis of variance (ANOVA) followed by Bonferroni post hoc test. All statistical analyses were performed on GraphPad Prism version 9.4.0 for Windows (GraphPad Software, San Diego, CA, USA). A value of *p* < 0.05 was considered to be statistically significant. 

## Data Availability

The data supporting this study’s findings are available from the corresponding author.
